# Evolution of Pediatric Urology at Sindh Institute of Urology and Transplantation

**DOI:** 10.3389/fped.2014.00088

**Published:** 2014-09-08

**Authors:** Sajid Sultan

**Affiliations:** ^1^Philip G. Ransley Department of Paediatric Urology, Sindh Institute of Urology and Transplantation (SIUT), Karachi, Pakistan

**Keywords:** pediatric urology, pediatric transplantation, pediatric urolithiasis, perinatal urology, pediatric urology in Pakistan

## Abstract

Sindh Institute of Urology and Transplantation was started in 1972 as an eight bedded department of genitourinary surgery in a government hospital by its pioneer and present director Syed Adib ul Hassan Rizvi. Responding to the socioeconomic dynamics and the needs of the patient population the facility grew into the largest tertiary care Urology, Nephrology, and Transplant center of south Asia. One of the salient components has been the evolution of the Department of Pediatric Urology, which in itself has shown a tremendous growth into an internationally recognized center for pediatric urology services taking care of all aspects including center of excellence for pediatric stone disease. The guiding mission of this institute remains to provide free medical services at zero cost without any discrimination to all who come to its doorstep and matching with high standard of care without compromising their dignity and self-respect. This institute highlights the fact that lack of resources is no excuse and is a role model for developing countries, where national and international support, motivation, and cooperation can offer more advanced and better quality medical services to our children.

## The SIUT

Pakistan is part of south Asia. Extending from the Himalayas in the north to the Arabian sea in the south, it has a population of about 184 million (1) of which approximately 36% are under the age of 14 years (2) and with a birth rate of nearly 4% producing almost 7 million newborns annually. There is an enormous demand for pediatric health care services from all disciplines. These facilities, including urology services, have always been inadequate for the adult population, let alone for children, in both the government and the private sectors. Overall, only 2% of GDP is spent on healthcare and the share of government spending is 0.4%.

Sindh Institute of Urology and Transplantation (SIUT) made a humble beginning in 1972 as a small 8-bed unit for genitourinary surgery in a large 1500 bed government hospital called Civil Hospital, Karachi. It was the brainchild of its present director Syed Adib ul Hassan Rizvi, who at that time had returned from United Kingdom as a young trained urologist. His vision and mission was to provide free medical services – without any discrimination whatsoever – to all who come to its doorstep and matching this with a high standard of care, and without compromising their dignity and self-respect. This remains the guiding mission for SIUT.

From the original 8 beds this unique foundation has expanded progressively so that today it has two 400 bed units including a dedicated oncology hospital and satellite units elsewhere in Karachi and in Sukkur. In 1991, it was upgraded to the status of an “Institute” with financial and functional autonomy through an Act of the Assembly and named “The SIUT.” In 2009, SIUT was granted independent degree awarding status by the Higher Education Commission of Pakistan.

Sindh Institute of Urology and Transplantation’s academic activities encompass training and education of all cadres of medical personnel. Expertise available in the Institute and collaboration with other national and international organizations has enabled SIUT to play a leading role in clinical postgraduate training and research in nephrology, urology, pediatric urology, GI and hepatology, and transplant sciences. A Centre for Biomedical Ethics and Culture (CBEC) was established in 2004, which now offers a Masters program in Bioethics. A 4-year training program for medical technologists in all disciplines at Zainul Abedin School of Medical Technology was inaugurated in 2005 under the supervision of the department of medical education.

Sindh Institute of Urology and Transplantation, at present, is the largest center of urology, nephrology, and transplantation in south Asia performing 750 dialysis sessions per day and has performed more than 4200 renal transplants. More than 200,000 patients attend outpatient clinics and an additional 100,000 are seen in the emergency department each year. All are seen and treated at zero cost and with no effort spared to provide the best possible treatments. The story of its origin, development, and current status is well told in the book by Ms. Zubeida Mustafa (3).

## Evolution of Pediatric Urology at SIUT

Children with urological and nephrological problems have always been part of the patient population since SIUT’s inception. However, with a growing patient population and expansion of the institute facilities, it became apparent that the needs and requirements of their management are much different from the adults and it became clear that if they were to be treated appropriately, then a dedicated pediatric facility had to be created. This has come about and matured through a series of happy coincidences and the dedicated work of a small group of people. At the forefront of these was Adib Rizvi himself who committed wholeheartedly to the concept and made it happen.

## The Early Years – Perinatal Urology and Stones

In 1991, Ms. Harjeet (“Jeeta”) Dhillon, a Perinatal Urologist from Great Ormond Street Hospital (GOS), London, UK, visited Karachi, Pakistan, with a delegation from the Royal College of Surgeons of England for a joint meeting with the College of Physicians and Surgeons of Pakistan where SIUT played a key role. She made a presentation about the Prenatal Diagnosis of hydronephrosis and congenital urinary tract abnormalities and inspired me, as a young resident with an interest in hydronephrosis, to establish a perinatal urology clinic at SIUT, which was the very first specialized pediatric urology clinic in Pakistan. This, in turn, led to a joint study with Dr. Shahida Zaidi and a retrospective analysis of 50,000 routine obstetric ultrasounds in a single center was presented in the National Nephrourology Meeting in Peshawar in 1994. However, even in a short visit, Ms. Dhillon recognized the importance of an institution like SIUT for Pakistan and the need for the development of pediatric urological services. She became a regular visitor for lectures, meetings, seminars, symposia, and workshops on perinatal urology involving obstetricians, radiologists, and obstetric ultrasonologists from all over the country.

In what is retrospectively a key moment in the development of pediatric urology at SIUT and at her initiative Ms. Dhillon organized a joint visit with Mr. Philip G. Ransley, a senior pediatric urologist also from GOS, for the first SIUT symposium in 1994 and the seeds of a dedicated pediatric service were sown.

**Figure 1:**
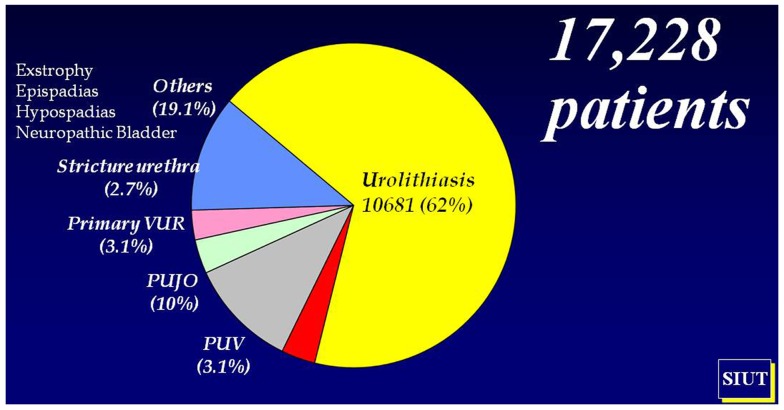
**Pediatric urological workload (1998–2013)**.

**Figure 2:**
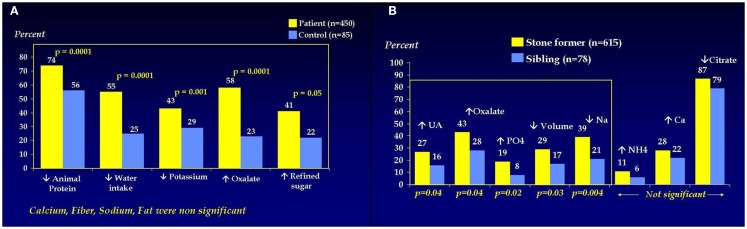
**(A)** Dietary risk factors in pediatric stone formers. **(B)** Urinary risk factors in pediatric stone formers.

The frequency of stone disease in Pakistan meant that the next step in establishing pediatric urology within SIUT was the setting up of a dedicated pediatric stone clinic and to this day urolithiasis comprises almost 60% of our workload (Figure 1) and calculus anuria is a common occurrence. Such difficult problems in small babies led to a parallel pediatric nephrology service being established, which in turn has grown along its own evolutionary path. With the nephrologists came nutritionists and the clinic now assesses dietary, metabolic, and urinary risk factors in ongoing studies (Figures 2A,B) (4–7).

The provision of all the necessary equipment for the endoscopic and radiological management of stones in children created a fully comprehensive pediatric stone service. This did not happen overnight but it cannot have happened so quickly or so smoothly without the generous input from Mr. Hugh Whitfield, urologist, and Dr. Michael Kellet, interventional radiologist, both from the Institute of Urology, London, UK, who visited regularly and contributed significantly to our efforts. Of course the clinic established primarily for stone disease rapidly delivered increasing numbers of patients with underlying urological anomalies for whom additional facilities were required (8, 9).

## General Pediatric Urological Services

As the service gathered momentum Professor Rizvi and the SIUT management invited me to accept responsibility for the pediatric urological services and if what follows seems a somewhat personal account, it is, I am afraid, necessarily so. Mr. Ransley and Ms. Dhillon remained very supportive and following a visit by Professor Rien Nijman in 2000, I left for a training position in his unit in Sophia Children’s Hospital in Rotterdam in 2000–2001. Apart from the routine spectrum of pediatric urological procedures, his unit was widely recognized as the shrine for pediatric urodynamics and we have tried as far as possible to transfer the lessons learned into our own pediatric urodynamic unit at SIUT.

In 2000, 2003, and 2005, I had opportunities to get exposed to a wider spectrum of pathology and its management at GOS in London courtesy Ms. Dhillon and Mr. Ransley. Association with a renowned group of specialists who were there as faculty and with observers from around the world introduced me and therefore SIUT to the “world” of pediatric urology. The basics of diagnosis by selective use of different imaging techniques and an analytical approach to treatment were the great lessons learned at GOS. Exposure to the specialist nursing care of pediatric urological patients, urodynamics, stoma care, and clean intermittent catheterization provided us with an understanding of how important these services are for the successful management of children with urinary tract anomalies and as far as possible we have attempted to implement a similar model at SIUT.

Following his retirement from GOS, Mr. Ransley’s more frequent visits contributed very significantly to the progress of the Pediatric Urology Department at SIUT and in 2004 a separate Pediatric Urology ward was created with 24 beds, which later got extended to 40 beds. It was subsequently dedicated appropriately as the Philip G. Ransley Department of Pediatric Urology in recognition of all the hard work and inspiration that had brought it into being. We are proud that the President and government of Pakistan also acknowledged the source of our progress by awarding him the Sitara-i-Imtiaz (Civil Award) in 2010.

The rest, as they say, is history. The workload in pediatric urology grew almost exponentially and in addition to the residents rotating from the urological training program, who now come for a mandatory 6-month exposure to pediatric urology, a 2-year fellowship training was inaugurated. The training is validated by an exit examination both written and clinical and the successful completion of a dissertation on a selected topic, which must include original work. The first three successful graduates from that fellowship initiative now form the key senior team running pediatric urology at SIUT. Four more candidates are at different stages of their fellowship training; prior to the formalization of this training program a great deal of work was done and much beneficial experience gained by pediatric surgeons and urologists who have returned to various institutions of the country to continue their work as pediatric urologists.

From a slow start the service expanded rapidly so that we now see up to 300 patients in a general clinic every Wednesday and a similar number in a stone clinic every Friday and the nephrological patients are seen separately every Tuesday. Waiting to be seen is an inevitability in such a clinic, particularly, given the nature and distribution of our patient base, we can never know which patients are coming until they arrive. Fortunately, our records system can handle this! The children have a musician and entertainers to occupy them and they are provided with sustenance. Each clinic brings with it the need for emergency admission for up to about 10 patients with massive tumors, acute retention, and calculus anuria being the commonest of these problems.

Fortunately, our ability to handle this workload is enabled by a superb team in the operating theaters who are themselves specialized so that cases for laparoscopy, PCNL, or reconstructive surgery can all run quite smoothly. Pediatric urology has three operating days per week every Monday, Thursday, and Saturday and we are fortunate that on these days we can utilize four operating tables simultaneously.

The list finishes only when the last patient is in recovery, which may often be late in the evening. In this way, we are able to handle 4000 admissions a year and 2500 surgical interventions.

The nature of Pakistan and our patient profile requires us to run a flexible service, which is always stretched to the limit and could not function without the huge support we receive from all departments in SIUT but particularly from senior faculty adult urology/adult and pediatric nephrology, laboratory, radiology, and anesthesia together with the technical staff in the operating theaters. A breakdown in Figure 1 shows the pattern of work from 1998 to 2013 and of course stones are dominant but the complex oncology and reconstructive surgery for traumatic urethroplasty, the neuropathic bladder, and exstrophy repair continues to increase year on year. We now have specialized staff in anesthesia and we can work comfortably in the neonatal period, which is essential in view of our origins as a prenatal service.

## Specialist Pediatric Urology Services

### Pediatric urolithiasis

As already mentioned urolithiasis constitutes more than 60% of our workload and the training acknowledged earlier was essential in developing our minimally invasive surgery (MIS) service. We have acquired the requisite expertise in MIS such as cystolithoclast, ureterorenoscopy, and percutaneous nephrolithotomy for children (10–12). More and more children with urolithiasis are being managed endoscopically with MIS and open surgery is in rapid decline.

### Perinatal urology

Within the general clinic there is a section dedicated to perinatal urology. The presence of an ultrasonologist (and an ultrasound machine!) in the clinic is of particular value for this group of patients and parents attending for prenatal counseling are also seen here. Our in-house ultrasound and nuclear medicine services are now adapted to the need of the newborn with an urinary tract abnormality detected by prenatal ultrasound.

### Pediatric uro-oncology services

Pediatric urological tumors have been a steadily increasing part of our workload and we have now treated more than 130 Wilms’ tumors with internationally comparable outcomes. We are able to undertake all the necessary staging imaging and investigations and we have to take the responsibility for the administration of chemotherapy and its complications. Fortunately, we are well supported by a visiting pediatric oncologist who holds a weekly clinic with us and there is a monthly tumor review meeting. Unfortunately, the often late presentations of massive tumors create significant medical and surgical challenges particularly with neuroblastoma and rhabdomyosarcoma.

### Pediatric renal transplant services

A living related renal transplant (LRRTx) program was started at SIUT, mainly for adults, in 1986. To date 4200 (mostly adult), LRRTx have been performed. Initially, the pediatric renal transplants were performed by the adult transplant team. However, with the establishment of pediatric urology and nephrology services, more and more end-stage renal disease patients were identified some of which also required lower urinary tract reconstruction prior to LRRTx. A separate pediatric program was started in 2005. A valuable contribution to the program by Dr. Oswald Fernando, pediatric transplant surgeon from UK and Dr. Chula Goonasekera, intensivist from Sri Lanka is well acknowledged. So far 605 children have had a successful LRRTx – the youngest being 2 years old. About 20 patients required augmentation cystoplasty prior to their transplants. Success rate has been more than 90% graft survival (13–15).

### Reconstructive surgery

Pakistan has an unenviable record for road traffic accidents and other forms of trauma and although being in the center of a city we do not often receive them acutely. There is a regular flow of cases with ruptured urethras who have often had indwelling suprapubic catheters for years following unsuccessful attempts at repair. More than 100 of these have been successfully restored to normal voiding. There is a large neuropathic population undergoing lower tract reconstruction and it has been pleasing the way that intermittent catheterization has been accepted even by the rural population once a cheap source of disposable catheters was identified. Some of these have been in renal failure and lower tract reconstruction has successfully preceded transplantation in a growing number of cases (15, 16). Surgery for hypospadias, epispadias, and bladder exstrophy is well established and Dr. Gianantonio Manzoni from the Policlinico in Milan has conducted a number of in-house workshops to great effect in order to keep the whole team abreast of modern techniques in penile reconstruction.

### Laparoscopy

Laparoscopic workshops were conducted in 2003 and 2004 by Mr. Imran Mushtaq, Consultant Pediatric Urologist from GOS and later by Mr. Ala El-Ghoneimi in 2010. We are in the early stages of developing laparoscopic services for children but laparoscopy for disorder of sexual development (DSD), orchidopexy, and nephrectomy are now routine with a recent shift to the extraperitoneal approach for the latter. A robot is not currently available to us but this is eagerly anticipated within a few years.

## Outreach

In keeping with the ethic of SIUT, treatment is provided free and with dignity. Many of our patients are very poor and often travel long distances from all parts of the country to seek treatment. As a consequence, it is necessary to tailor our treatment protocols and programs to try and minimize the cost and disruption of travel. This is of course an important factor with which we must juggle and balance with safe follow up and our social workers are always busy! Apart from the long term investment in the fellowship program, in order to try and encourage the development of pediatric urology services in other parts of the country we have conducted three live surgical workshops in 2009, 2010, and 2012. Apart from Mr. Ransley and Ms. Dhillon, the guest faculties have included world renowned pediatric urologists, namely, Dr. Ricardo González, his wife Professor Dr. Barbara Ludwikowski, Dr. Gianantonio Manzoni, Mr. Padraig (Pat) Malone, Professor Alaa El-Ghoneimi, Dr. Richard Hurwitz, and Ms. Stephanie Warne. We are enormously grateful to them all for their willingness to come to Karachi and to work so hard in order to help pediatric surgeons and urologists from all over the country to acquire the latest concepts and techniques in pediatric urology. To that we must add the already mentioned workshops conducted by Dr. Manzoni and the recent teaching visit by Professor Faisal Ahmed from Glasgow focusing on DSD and endocrinology.

Their influence has not been in vain and we have increasingly made presentations at scientific meetings such as the American Academy of Pediatrics and the European Society for Pediatric Urology where we were honored to receive the first prize in the clinical poster section in Amsterdam in 2009. Even with the constraints of time due to enormous patient load more and more effort is being made by the team to contribute to the scientific publications in peer reviewed journals.

## The Way Ahead

We, who are part of the Department of Pediatric Urology, draw inspiration and confidence from the evolution of our mother organization, the SIUT, and that of our own department. Where it has been shown to such an incredible extent that lack of resources is no excuse. A full-fledged pediatric urology and nephrology hospital is planned to be functional in 5–7 years. Besides building a state of the art facility this will also involve the most important task of building a highly dedicated and expert team of medical and administrative personnel on the SIUT model with friends and colleagues worldwide ensuring that we deliver the best possible care.

## Conflict of Interest Statement

The author declares that the research was conducted in the absence of any commercial or financial relationships that could be construed as a potential conflict of interest.

## Supplementary Material

The Supplementary Material for this article can be found online at http://www.frontiersin.org/Journal/10.3389/fped.2014.00088/abstract
