# Supplementary material for: Evolution of Pediatric Urology at Sindh Institute of Urology and Transplantation
Source: Front Pediatr. 2014 Sep 8;2:88. doi: 10.3389/fped.2014.00088 (PMC4157550; doi:10.3389/fped.2014.00088)
Supplement: Supplementary file 1 [file Presentation1.PDF]

Figure 1: Senior faculty members of SIUT

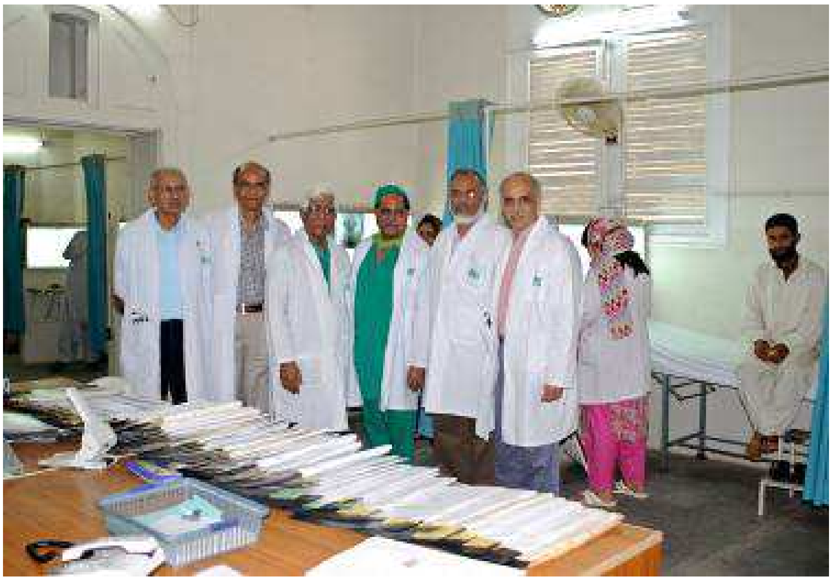

Dr Fazal Akhtar, Dr Anwar Naqvi, Dr Adib Rizvi (Director), Dr Zafar Hussain, Dr Altaf Hashmi and Dr Ejaz Ahmed in outpatient clinic

Figure 2: Fellows in Paediatric Urology

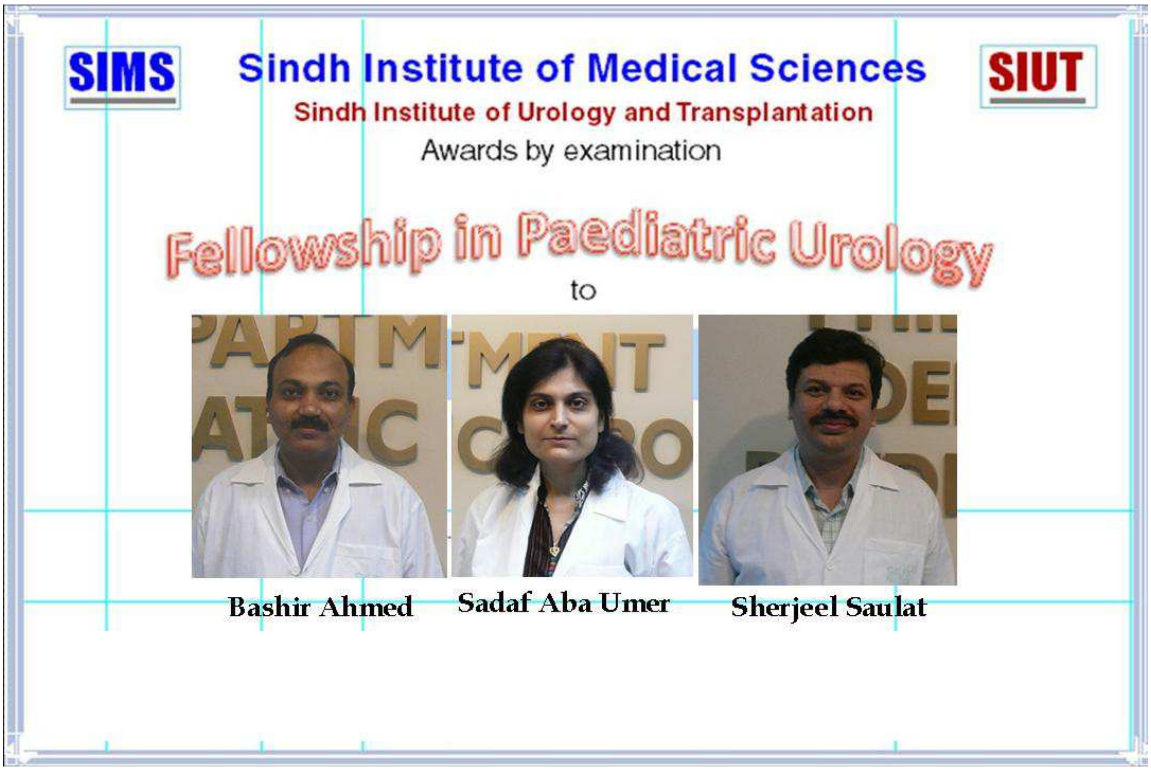

Figure 3: Philip G Ransley Department of Paediatric Urology

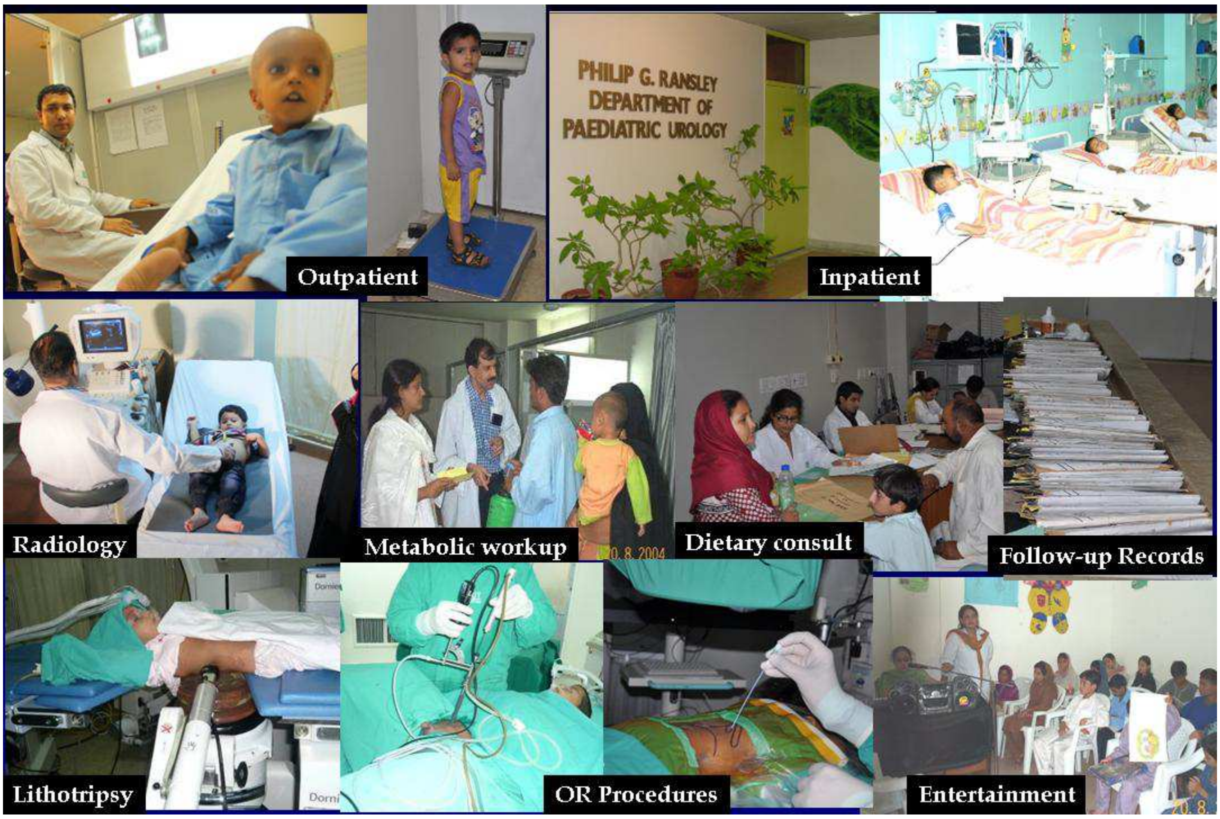

Figure 4: International Visiting Faculty at SIUT

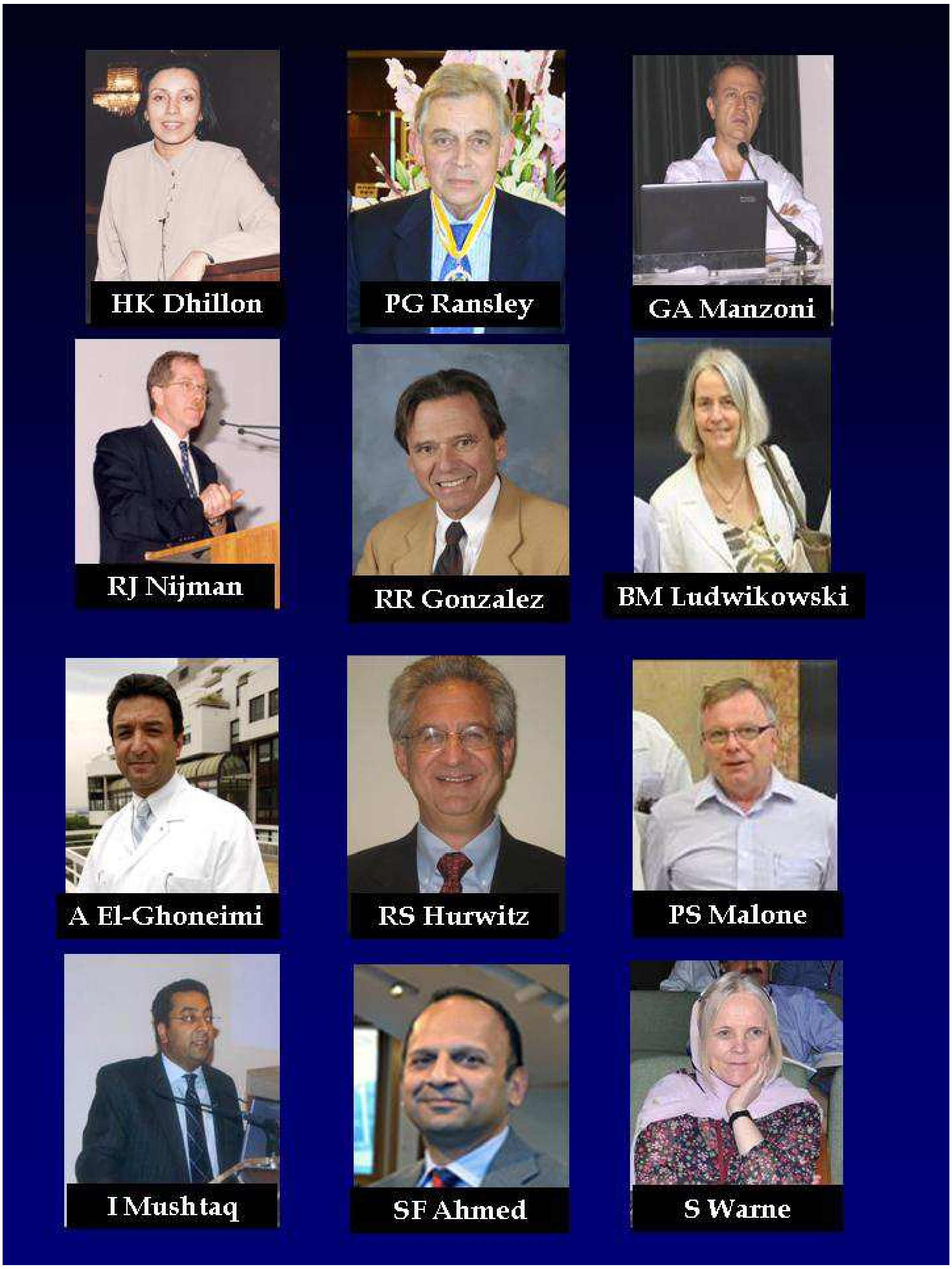

Table 1: Minimally Invasive Surgery (MIS) for Stone Disease

n = 6088

• Extracorporeal Shockwave lithotripsy (ESWL)	1534
• Percutaneous Nephrolithotomy (PCNL)	1627
• Ureterorenoscopy + Lithotripsy (URS + LC)	1317
• Perurethral / Percutaneous Lithotripsy (PUCL/PCCL)	1610

Table 2: SIUT presentations at international conferences

- Endoscopic management of urolithiasis in children.
  Sajid Sultan, Anwar Naqvi, Adib Rizvi
  Annual ESPU Congress, Madrid, Spain 2003
- Clean intermittent Catheterization (CISC) in Children through Continent Catheterizable channel: A developing country Experience.

  Sajid Sultan, Ijaz Hussain, Bashir Ahmed, Sadaf Aba Umer, Sharjeel Saulat, Aamir Mehmood, Anwar Naqvi, Adib Rizvi.
  American Academy of Pediatrics Section on Urology Meeting, October 27-29, 2007, San Francisco, California. (2007) [POS 20]
- Urinary Crystallization index: A risk marker for Calcium Oxalate lithiasis.
  Bashir Ahmed, Sajid Sultan, Ijaz Hussain, Sadaf Aba Umer, Sharjeel Saulat, Aamir Mehmood, Mirza Naqi Zafar, Anwar Naqvi, Adib Rizvi.
  19th Annual ESPU Congress, Nice, France 2008 JPUrol 2008 Apr; 4 (1 suppl): S26.
- Pediatric urolithiasis with renal failure: SIUT experience.
  Naqvi SA, Sultan S, Ijaz H, Ahmed B, Umar SA, Saulat S, , Zafar MN , Rizvi SA.
  19th Annual ESPU Congress, Nice, France 2008 JPUrol. 2008 Apr; 4(1 suppl): S22.
- Percutenous Nephrolithotomy (PCNL) in Paediatric Stones.
  Sultan S, Ijaz H, Ahmed B, Umar SA, Saulat S, Mehmood A, Naqvi SA, Rizvi SA.
  19th Annual ESPU Congress, Nice, France 2008 JPUrol. 2008 Apr; 4 (1 suppl): S21.
- Endoscopic Management pf Paediatric Ureteric Calculi by Holmium:Yag Laser Lithotripsy.
  Ijaz Hussain, Sajid Sultan, Bashir Ahmed, Sadaf Aba Umer, Sherjeel Saulat, Aamir Mehmood, Raees Taqvi, Anwar Naqvi, Adib Rizvi.
  AAP Section on Urology & Pediatric Urology Nurse Specialists (PUNS), International Children's Continence Society (ICCS), Society for Fetal Urology (SFU). October 10-13, 2008, Boston, MA
- Endoscopic management of Paediatric Ureteric Calculi by Holmium:Yag laser lithotripsy.
  Saulat S, Sultan S, Ijaz H, Ahmed B, Umar SA, Taqvi R, Mehmood A, Naqvi SA, Rizvi SA.
  20th Annual ESPU congress, Amsterdam, The Netherlands 2009. JPUrol. 2009 Apr; 5 (1 suppl): S36.
- Composition of renal and bladder calculi in pediatric stone formers.
  Umar SA, , Sultan S, Zafar MN, Ijaz H, Ahmed B, Saulat S, Taqvi R, Mehmood A, Naqvi SA, Rizvi SA.
  20th Annual ESPU congress, Amsterdam, The Netherlands 2009. JPUrol. 2009 Apr; 5 (1 suppl): S32.
- Renal Transplant in children with Lower Urinary Tract Dysfunction: A Developing Country Experience.
  Sultan S, Ijaz H, Ahmed B, Umar SA, Saulat S, Naqvi SA, Rizvi SA.

21st Annual ESPU Congress, Antalya, Turkey 2010. JPUrol 2010 Apr; 6 (1 suppl): S51.

- Percutaneous Nephrolithotomy (PCNL) in Paediatric Stones: A Single Centre Experience.
  Sultan S, Ijaz H, Ahmed B, Umar SA, Saulat S, Naqvi SA, Rizvi SA.
  21st Annual ESPU congress, Antalya, Turkey 2010 . JPUrol. 2010 Apr; 6 (1 suppl): S34-35.
- Clean Intermittent Catheterization (CIC) in Children Through Continent Catheterizable Channel: A Developing Country Experience.
  Iaz Hussain, Sajid Sultan, Bashir Ahmed, Sadaf AbaUmer, Sherjeel Saulat, Anwar Naqvi, Adib Rizvi.
  21st Annual ESPU congress, Antalya, Turkey 2010
- Challenges and Out come of Wilms' Tumour: A Developing Country Experience.
  Sadaf Aba Umer, Sajid Sultan, Bashir Ahmed, Sherjeel Saulat, Anwar Naqvi, Adib Rizvi.
  22nd Annual ESPU Congress-Copenhagen, Denmark-2011. S18-3 (PP)
- Open Surgical Management of Paediatric Urolithiasis: A Developing Country Perspective.
  Sajid Sultan, Bashir Ahmed, Sherjeel Saulat, Sadaf Aba Umer, Anwar Naqvi, Adib Rizvi
  23rd Annual ESPU Congress - Zurich, Switzerland - 2012 S11: Stone
- Diagnostic Complexities of genitourinary tuberculosis (GUTB) in children: A Developing country experience
  Sajid Sultan, Bashir Ahmed, Sherjeel Saulat, Sadaf Aba Umer, Anwar Naqvi, Adib Rizvi
  23rd Annual ESPU Congress - Zurich, Switzerland – 2012, S13: Miscellaneous
- Percutaneous Nephrolithotomy (PCNL) In Paediatric Stones.
  Sajid Sultan, Bashir Ahmed, Sherjeel Saulat, Sadaf Aba Umer, Anwar Naqvi, Adib Rizvi
  23rd Annual ESPU Congress - Zurich, Switzerland - 2012 S11: Stone
- "Paediatric Urolithiasis: Composition Assessment on Non Contrast Ct Scan.
  Sajid Sultan, Sadaf Aba Umer, Sherjeel Saulat, Bashir Ahmed, Kashifuddin Qayoom Soomro, Mirza Naqi Zafar, Shabbir Naeem, HK Dhillon, Philip G Ransley, Anwar Naqvi, Adib Rizvi
  24th Annual ESPU Congress, Genoa Italy. 2013
- Paediatric Renal Tumours: A Developing Country Experience.
  Sajid Sultan, Sherjeel Saulat, Sadaf Aba Umer, Bashir Ahmed, Kashifuddin Qayoom Soomro, Shamvil Ashraf, Muhammad Mubarak, HK Dhillon, Philip G Ransley, Anwar Naqvi, Adib Rizvi
  24th Annual ESPU Congress, Genoa Italy. 2013

Table 3: SIUT publications in peer reviewed journals

- Naqvi A, Rizvi A. Pediatric nephrolithiasis in Pakistan. J Pak Med Assoc 1982; 32:177.
- Naqvi A, Rizvi A, Syed SJ. Bladder stone disease in children: clinical studies. J Pak Med Assoc 1984; 34:94.
- Naqvi A, Rizvi S, Syed SJ. Role of infection in bladder stone disease in children. J Pak Med Assoc 1984; 34:132.
- Rizvi A, Naqvi A, Hussain Z. Renal stones in children in Pakistan. Br J Urology 1985; 57:618-21.
- Rizvi SA. Pediatric renal transplantation in Pakistan. Transplant Proc 2000 May;32(3):652-3
- Rizvi, A. Naqvi, Z. Hussain, M. Hussain, A. Hashmi, F. Akhtar, M.N. Zafar, E. Ahmed, S. Sultan, S. Aziz, A. Shehzad, R. Khalid. Why is it more difficult to transplant children? A perspective in developing countries. Transplant Proc 2001 February; 33(1-2):1742-43
- S. A. H. Rizvi, S. A. A. Naqvi, Z. Hussain, A. Hashmi, M. Hussain, M. N. Zafar, S. Sultan, H. Mehdi. Paediatric urolithiasis: Developing world perspectives. J Urol 2002 168:1552-1525.
- S. A. H. Rizvi, S. A. A. Naqvi, Z. Hussain, A. Hashmi, M. Hussain, M. N. Zafar, S. Sultan, H. Mehdi. The Management of Paediatric Urolithiasis in Pakistan: An Experience of 1440 children. J Urol 2003 169:634-7
- Rizvi SA, Sultan S, Zafar MN, Ahmed B, Faiq SM, Hossain KZ, Naqvi SA. Evaluation of children with urolithiasis. Indian J Urol. 2007 Oct;23(4):420-7.
- Sultan S, Hussain I, Ahmed B, Aba Umer S, Saulat S, Naqvi SA, Rizvi SA. Clean intermittent catheterization in children through a continent catheterizable channel: a developing country experience. J Urol. 2008 Oct;180(4 Suppl):1852-5
- Rizvi SA, Sultan S, Ijaz H, Mirza ZN, Ahmed B, Saulat S, Umar SA, Naqvi SA. Open surgical management of pediatric urolithiasis: A developing country perspective. Indian J Urol. 2010 Oct;26(4):573-6.
- Rizvi SA, Sultan S, Zafar MN, Naqvi SA, Lanewala AA, Hashmi S, Aziz T, Hassan AS, Ali B, Mohsin R, Mubarak M, Farasat S, Akhtar SF, Hashmi A, Hussain M, Hussain Z. Pediatric kidney transplantation in the developing world: challenges and solutions. Am J Transplant. 2013 Sep;13(9):2441-9.
